# The Impact of *Angiotensin-Converting Enzyme 2* (*ACE2*) Expression Levels in Patients with Comorbidities on COVID-19 Severity: A Comprehensive Review

**DOI:** 10.3390/microorganisms9081692

**Published:** 2021-08-09

**Authors:** Rui Rodrigues, Sofia Costa de Oliveira

**Affiliations:** 1 Department of Pathology, Division of Microbiology, Faculty of Medicine, University of Porto, Al. Hernâni Monteiro, 4200-319 Porto, Portugal; seabra.rodrigues.rui@gmail.com; 2 Center for Research in Health Technologies and Information Systems (CINTESIS), R. Dr. Plácido da Costa, 4200-450 Porto, Portugal

**Keywords:** ACE2, SARS-CoV-2, COVID-19, comorbidity, viral receptor

## Abstract

Angiotensin-Converting Enzyme 2 (ACE2) has been proved to be the main host cell receptor for the binding of the severe acute respiratory syndrome coronavirus 2 (SARS-CoV-2), the virus responsible for the COVID-19 pandemic. The SARS-CoV-2 spike (S) protein binds to ACE2 to initiate the process of replication. This enzyme is widely present in human organ tissues, such as the heart and lung. The pathophysiology of ACE2 in SARS-CoV-2 infection is complex and may be associated with several factors and conditions that are more severe in COVID-19 patients, such as age, male gender, and comorbidities, namely, cardiovascular diseases, chronic respiratory diseases, obesity, and diabetes. Here we present a comprehensive review that aims to correlate the levels of expression of the *ACE2* in patients with comorbidities and with a poor outcome in COVID-19 disease. Significantly higher levels of expression of *ACE2* were observed in myocardial and lung tissues in heart failure and COPD patients, respectively. An age-dependent increase in SARS2-CoV-2 receptors in the respiratory epithelium may be also responsible for the increased severity of COVID-19 lung disease in elderly people. Although the role of ACE2 is highlighted regarding the damage that can arise upon the SARS-CoV-2 invasion, there was no association observed between renin-angiotensin-aldosterone system (RAAS) inhibitors and the severity of COVID-19.

## 1. Introduction

In December 2019 in Wuhan, China, a novel coronavirus was detected: the severe acute respiratory syndrome coronavirus 2 (SARS-CoV-2). This virus was responsible for the Coronavirus Disease 2019 (COVID-19) and has spread across the globe, being classified as a global pandemic in March 2020 [1]. Up to date, more than 180 million confirmed cases and almost 4 million deaths have been reported to the World Health Organization (WHO) [2].

Human-to-human transmission is well established for this virus, which spreads through direct contact or aerosolized nasal droplets [3,4,5,6]. The viral entry through the respiratory tract is thought to be the major route of infection [7].

The clinical spectrum of the infection may range from asymptomatic to critically ill pneumonia associated with acute respiratory distress syndrome (ARDS) [8]. The main symptoms displayed by COVID-19 patients are fever, cough, dyspnoea, and myalgia or fatigue, indicating that SARS-CoV-2 primarily infects the respiratory tract [9]. However, SARS-CoV-2 infection may result in symptoms associated with other tissues from the digestive, nervous, and cardiovascular systems [10,11,12,13,14].

SARS-CoV-2 uses Angiotensin I Converting Enzyme 2 (ACE2) for entry into the host cells by a mechanism that requires multiple factors [15]. ACE2 has a wide distribution across human tissues, including the lung, heart, liver, stomach, ileum, colon, and kidney, indicating that SARS-CoV-2 may infect multiple organs, explaining the positive detection of SARS-CoV-2 in patients’ faeces and urine [16,17,18,19].

This comprehensive review describes the current knowledge regarding the correlation between the levels of expression of the ACE2 in various organs and poor outcomes in COVID-19 patients. PubMed was used as the electronic database for our literature research which was carried out from December 2019 to February 2021. The search terms used were: (SARS-CoV-2 OR “new coronavirus” OR COVID-19) AND “ACE2 expression” AND (“severity” OR “ICU”). Hence, the query identified 62 articles. These were only considered if written in English. Two were duplicated and 23 were excluded for not addressing the topic or not fitting the question.

## 2. Viral Pathogenesis—ACE Receptors and Co-Receptors

The virus transmembrane spike glycoprotein (S protein) is essential for binding to the cellular membrane ACE2 and for the attachment of the virus to the target cells. Based on modelling and docking, it was discovered that the residues 441Leu, 72Phe, 479Gln, 480Ser, 487Asn, and 491Tyr of the mature S protein appear to be critical for binding to the human ACE2 receptor and determining the host cellular range [16].

Upon binding to ACE2, the S protein requires cleavage by proteases, such as transmembrane protease serine 2 (TMPRSS2), readily expressed in lung tissue, to fuse with the cell membrane, resulting in SARS-CoV-2 entry and replication in the target cells [20,21]. Despite being the most relevant S protein cleavage protease, the viral receptor and co-receptors should be co-expressed on the same cell to mediate cell entry. However, TMPRSS2 may not be present ubiquitously in every human tissue; thus, the priming of the S protein may depend on other host proteases [22]. As an example, *ACE2* expression in the adult human heart is higher than that in the lung (adjusted *p* < 0.0001), but the low percentage of ACE2+/TMPRSS2+ cells in heart tissue would prevent its vulnerability to SARS-CoV-2 [23]. Hereupon, Liu et al. found out that proteases cathepsin L (CTSL) and FURIN (furin; paired basic amino acid cleaving enzyme) were expressed in the adult heart at a similar level to that in the lung, compensating for TMPRSS2 and mediating cardiac involvement in COVID-19 [23].

Over the last months, three new variants of concern, the B.1.1.7 (United Kingdom), B.1.351 (South Africa), and P.1 (Brazil) have emerged and have had an impact upon transmissibility, virulence, and immune and neutralizing antibody escape [24,25,26,27]. All the SARS-CoV-2 variants reported to date have had multiple mutations in the spike (S) protein, specifically in the receptor-binding domain (RBD) [28,29]. Verma et al. reported some critical mutations that could increase the binding affinity of the SARS-CoV-2 RBD with ACE2, increasing the viral infectivity and pathogenicity [27]. The hotspot stabilizing residue mutations N501I, N501Y, Q493L, Q493H, and K417R, strengthen the RBD–ACE2 complex by modulating the interaction statistics at the interface [27]. Understanding the effect of these mutations will help to develop potential vaccines and to outline therapeutic options.

### 2.1. Physiological Role of ACE2 Protein

ACE2 plays a pivotal role in the renin-angiotensin-aldosterone system (RAAS). Angiotensinogen, produced by the liver, is cleaved by renin, resulting in the formation of angiotensin I (Ang I). Subsequently, ACE is one of the enzymes that catalyses the conversion of Ang I to angiotensin II (Ang II) [30]. Ang II, the main active RAAS component, exerts its effects mainly via angiotensin-II type 1 receptors (AT1R). The major effects of Ang II include vasoconstriction, renal sodium reabsorption and potassium excretion, aldosterone synthesis, blood pressure elevation, and the induction of inflammatory and pro-fibrotic pathways [31,32].

During hypoxia, Ang II-induced pulmonary vasoconstriction aims to restore the ventilation–perfusion mismatch but simultaneously induces adverse pro-fibrotic effects [33]. Upon binding to AT1R, Ang II can induce reactive oxygen species (ROS) production by stimulating the activity of nicotinamide adenine dinucleotide (NADH) and nicotinamide adenine dinucleotide phosphate (NADPH) oxidases in vascular smooth muscle cells, promoting oxidative stress and vascular inflammation, which can lead to vascular injury and thrombus formation [34].

ACE2 is a transmembrane zinc metallocarboxypeptidase that counterbalances ACE by cleaving Ang II into Ang-(1-7) and Ang I into Ang-(1-9) [35], which is in turn converted into Ang-(1-7) by ACE [36]. Finally, Ang-(1-7) exerts vasodilating, anti-inflammatory, and anti-fibrotic effects through binding to the Mas receptor [36]. Thus, ACE2 facilitates the attendant cardioprotective actions of nitric oxide release, vasodilation, and the blunting of inflammation [37]. The ability of ACE2 to provide a negative regulation of Ang II and the receptor is of biological significance in pathological conditions where RAAS may be dysfunctional [35].

### 2.2. ACE2 Down-Regulation and Viral Infection

Viral binding to the receptor, with subsequent internalization via a clathrin-mediated endocytosis and the activation of proteolytic cleavage, leads to a loss of ACE2 function in the cell membrane [38,39,40]. The virological characteristics of SARS-CoV-2 are closely related to those of SARS-CoV-1, a coronavirus that caused the SARS epidemic in China, in 2002–2003. Both use the same receptor for viral cell entry [41,42]. After SARS-CoV-1 infection in mice, it was proved that lung ACE2 protein levels were greatly reduced, while ACE levels did not change [43]. These findings were consistent with the previous observation that coronaviruses specifically downregulate *ACE2* expression in host cells, depending on virus replication [21].

At first sight, one may speculate that SARS-CoV-2-induced down-regulation of *ACE2* may result in a decreased opportunity for further viral cell entry, thereby limiting viral spread. However, the binding affinity of SARS-CoV-2 to ACE2 is 10 to 20-fold stronger than the binding affinity of SARS-CoV-1 to the same receptor and this down-regulation would not be the reason for the decreased viral spread [44,45,46]. On the other hand, decreased ACE2 after viral invasion could impair the clearance of Ang II and hence lead to the aggravation of tissue damage, as discussed previously. 

In animal models, the loss of ACE2 was linked to decreased cardiac contractility and microcirculatory dysfunction and the administration of recombinant ACE2 was shown to inhibit the angiotensin II effects on the transforming growth factor (TGF)-β1 activation and collagen production and to attenuate whole aspects of pulmonary artery hypertension pathophysiology [47,48,49]. Indeed, the harmful effect of Ang II was previously demonstrated in several animal models of ARDS [50,51,52,53]. Therefore, *ACE2* down-regulation stimulated with the viral invasion was found to be one of the critical factors in the pathogenesis of SARS-CoV-2 infection, particularly in patients with threshold ACE2 deficiency (old age population, hypertensive patients, or with diabetes or prior cardiovascular diseases) [44,46].

## 3. ACE2 Expression Levels

After discussing the intimate relation between ACE2 and SARS-CoV-2 infection and the pathophysiology involved, we wondered if the levels of *ACE2* expression in the organism prior to the viral infection could be related to the severity of the disease and its outcomes. In fact, patients with more severe COVID-19 have higher viral loads in the respiratory tract (throat, bronchoalveolar lavage fluid, or sputum) and longer viral persistence than those who experience milder disease [54,55,56]. Not surprisingly, expression levels of the viral host receptor ACE2 and cell entry-associated molecules (e.g., TMPRSS2) are thought to be important and relevant factors influencing viral loads and infection [39,57,58]. What is known so far is that specific groups of patients are at an increased risk of more severe symptoms. Recent studies of the epidemiological characteristics of COVID-19 patients have revealed that severe infection is more likely in patients with an existing chronic medical condition [59]. Risk factors for an unfavorable outcome include older age, male gender, and underlying comorbidities such as obesity, hypertension, cardiovascular disease, diabetes, or chronic respiratory disease [60,61].

More precisely, in several large cohort studies, the clinical prevalence of comorbidities among confirmed COVID-19 patients ranged between 8.3% and 10.5%: 7.3% for diabetes mellitus, 6.5% to 15.0% for hypertension, 2.5% for coronary artery diseases, and 1.4% for cerebrovascular disease [1]. Among intensive care unit-admitted COVID-19 patients, a high prevalence of at least one comorbidity was described, ranging from 68% (Italian cohort) to 72% (Chinese cohort) [60,62,63].

Although some studies did not find differences in the frequency of diabetes or hypertension when compared to age-matched controls, in patients with severe COVID-19 (who required mechanical ventilation and/or intensive care unit (ICU) supportive care), a multivariate logistic regression showed that hypertension (odds ratio (OR) = 2.26, 95% confidence interval (CI), 1.12–4.63; *p* = 0.02;) and male gender (OR = 3.15, 95%CI, 1.56–6.66; *p* = 0.002;) remained as independent significant predictors of severity [64].

Cancer patients are highly susceptible to infections in general, and consequently are more vulnerable to SARS-CoV-2. The extensive immunosuppressive therapy due to chemotherapy or radiotherapy predisposes cancer patients to be at a higher risk of developing severe and critical consequences from COVID-19, including ARDS, septic shock, and acute myocardial infraction [65]. However, mortality from COVID-19 in cancer patients appears to be principally driven by age, gender, and preexisting comorbidities such as cardiovascular diseases, COPD, and hypertension [65,66]. In a prospective cohort study involving 800 patients with cancer and symptomatic COVID-19, Lee et al. found that immune-checkpoint inhibitors-immunotherapy (ICI-immunotherapy), hormone therapy, targeted therapy, and radiotherapy received in the 4 weeks before the COVID-19 diagnosis had no incidence on the overall rate of death [65]. Although few data are available on cancer patients affected by COVID-19 and treatment with ICIs, some authors have suggested that they might be beneficial in COVID-19-positive cancer patients by exerting and restoring cellular-mediated immunocompetence [67,68].

Taking these facts into account, we used the presence of these comorbidities as our variable to see if the levels of *ACE2* expression in the mentioned groups differed from the levels in the population without those risk factors and if that could be the explanation for the different presentation of the disease (Table 1).

### 3.1. Cardiovascular Disease

Despite the low expression in cardiomyocytes, *ACE2* is highly and specifically expressed in pericytes [16]. In a meta-analysis, the prevalence of cardiac injury in COVID-19 patients varied from 2% (95%CI, 0–5%) in non-ICU patients to 59% (95%CI, 48–71%) in non-survivors [23]. Thus, SARS-CoV-2 infection in the human heart might attack pericytes and cause the dysfunction of the capillary endothelial cells, inducing micro-circulation disorder [16]. Even though it has a beneficial role in cardiovascular function, *ACE2* expression in the heart tissue is elevated in several cardiovascular pathologies, such as ischemic heart failure, idiopathic dilated cardiomyopathy, and pulmonary hypertension [47,69,70]. A previous work showed that patients with heart failure had higher ventricular *ACE2* expression levels and a recent study with RNA sequencing revealed that myocardial *ACE2* expression was significantly increased in patients with heart failure (fold change = 3.00, *p* < 0.0001), which was further validated at the protein level by proteomics profiling (fold change = 1.82, *p* < 0.0001) [16,71].

These results indicate that heart failure patients are more susceptible to heart infection by SARS-CoV-2 and might develop further cardiac injury and even progress to a critical condition.

### 3.2. Pulmonary Diseases: Chronic Obstructive Pulmonary Disease (COPD) and Lung Cancer

Pinto et al. analyzed over 700 lung transcriptome samples of patients with comorbidities associated with severe COVID-19 and found that *ACE2* was highly expressed in these patients compared to control individuals [59]. More particularly, in patients with COPD, a lung RNA-seq dataset showed a significant upregulation in the expression of ACE2 in comparison to subjects with normal spirometry (controls) (*p* = 0.00034) and *ACE2* was significantly up-regulated in six out of seven lung transcriptome studies, suggesting that patients who have COPD or Pulmonary Arterial Hypertension (PAH) may have a higher risk of developing severe COVID-19 [59]. Besides, the staining of lung tissue sections from adults with PAH, a possible complication of COPD, has revealed increased ACE2 protein in the endothelium of pulmonary arteries, compared to healthy controls [77].

More studies support the fact that smokers and those with COPD have increased airway expression of *ACE2*, explaining the increased risk of severe COVID-19 in these subpopulations and highlighting the importance of smoking cessation [72,73,74,76]. This matches the results obtained in animal models, wherein exposure to smoke led to the upregulation of both the activity and the expression of *ACE2* in the airways [75,79]. However, regarding active smoking, evidence may be controversial and we should mention the hypothesis that nicotine may upregulate ACE but downregulate the compensatory *ACE2*/Ang-(1-7) receptor axis, and a preliminary meta-analysis indicated that active smoking was not associated with severity of coronavirus disease [99,100].

Cancer is also listed as a relevant co-morbidity and *ACE2* expression was found to be upregulated in renal cancer, gastrointestinal tumors, and lung cancer [78]. More particularly, a RNA-seq data of patients with lung adenocarcinoma showed that *ACE2* was expressed in 59% of cancer samples and only in 25% of adjacent lung normal samples and, among the samples expressing *ACE2*, the level of the gene was higher in the cancer, compared to the adjacent normal lung tissue (*p* = 0.0012) [59].

The mechanisms by which *ACE2* is up-regulated in patients with comorbidities associated with COVID-19 severity are not entirely understood, but genes associated with the epigenetic regulation of gene transcription, such as *HAT1* and *HDAC2*, were positively correlated with ACE2 [59]. These modulate chromatin and DNA condensation by changing the histone acetylation status, thus permitting gene transcription. This could be happening in lung tissue, facilitating *ACE2* expression, as observed during lung cancer and COPD [59].

### 3.3. Cancer

The hypothesis that the worst outcomes in patients with cancer and COVID-19 disease may be influenced by the fact that cancer tissues themselves might have a higher expression of viral entry related genes has attracted a lot of attention by the scientific community.

In a retrospective study, Ravaioli and colleagues investigated *ACE2* and *TMPRSS2* expression as susceptibility factors to SARS-CoV-2 infection [101]. Significantly higher *ACE2* expression was found in cancers of the adrenal gland, brain, colon, kidney, lung, pancreas, and stomach as compared with the relative healthy tissue, resulting in a likely higher susceptibility of these tumors to SARS-CoV-2 infection [101,102,103]. In an integrated analysis of *ACE2* and *TMPRSS2* gene expression across clinical, genetic, and microbiome domains, Bao et al. found that cancer tissues broadly have a lower expression of *ACE2* and *TMPRSS2*, though the cancers of the digestive tract do have the highest relative level [104]. Regarding the host immunity associations, the authors observed a strong correlation between *ACE2* expression levels and multiple immune gene signatures such as interferon-stimulated genes and the T cell-inflamed phenotype [104]. Higher *ACE2* levels in T cell-inflamed tumors may be relevant to the administration of cancer immunotherapy during the COVID-19 pandemic, especially in patients with tumors of the aerodigestive tract such as head and neck, lung, and colorectal/anal tracts. *TMPRSS2* was less associated with immune gene expression but was strongly associated with epithelial cell abundance [104]. The mechanisms of *ACE2* and *TMPRSS2* regulation could be targeted for preventive and therapeutic purposes in the whole population and especially in cancer patients. However, special attention should be given to the possible treatment with ACE2 inhibitors of COVID-19 patients with cancer who are receiving immunotherapy. This is since an increase in *ACE2* expression may be associated with a higher rate of response to ICIs [102,103]

### 3.4. Asthma

To approach this topic, we should first understand that at the beginning of the pandemic, asthma was thought to be a risk factor for COVID-19 severity, as it tends to exacerbate during respiratory viral infections [85]. Nonetheless, recent literature has shown the opposite, and Kimura et al. demonstrated a lower *ACE2* expression in the nasal epithelial cells of participants with asthma and allergic rhinitis as compared to healthy participants [86]. IL-13 is a cytokine associated with allergic asthma (type 2) and it was observed that IL-13 decreases *ACE2* expression in the nasal and airway epithelial cells [86]. Hence, reduced *ACE2* expression in patients with asthma is associated with reduced COVID-19 severity, the opposite of what was initially expected.

### 3.5. Obesity

Obesity is one of the main risk factors for COVID-19 critical illness leading to ICU admissions or death. Epidemiological data reported that these patients (Body Mass Index (BMI) > 35) have more than a seven-fold risk of being admitted to the ICU as compared with those with a BMI < 25 kg/m^2^. Moreover, patients with metabolic syndrome are highly susceptible to SARS-CoV-2 infection [80,105,106]. However, our literature search did not find as much evidence regarding obese patients and *ACE2* expression as one would have expected.

Nevertheless, it is still relevant to state that enhanced *ACE2* expression, pre-existing endothelial dysfunction, and the procoagulant state induced by adipocytokines dysregulation in metabolic syndrome may play a crucial role for the development of severe COVID-19 [82]. Moreover, the gene expression profile in human visceral and subcutaneous adipose tissues has demonstrated a higher *ACE2* gene expression compared to human lung tissue [80].

In fact, an increased *ACE2* expression in COPD patients who are overweight compared to those who are not overweight was observed and studies in mice showed that obese male mice fed a High Fat Diet displayed a significantly elevated expression of *ACE2* in the lung and trachea relative to chow-fed lean male mice [7,81].

### 3.6. Diabetes

Patients with diabetes mellitus are at higher risk of COVID-19 and developing severe symptoms that are often fatal [107]. ACE2 protein expression analyzed by immunohistochemistry in bronchial and alveolar samples displayed a significant increase in type 2 diabetic patients compared to the control group [84].

Furthermore, microarray and RNA-sequencing expression data showed that the expression of *ACE2* in pancreatic islets was significantly (*p* ≤ 0.05) increased in diabetic patients compared to non-diabetic patients and that *TMPRSS2* expression was positively correlated with hemoglubin A1c (HbA1c) levels (*p* = 0.04) [83]. Curiously, in this same study, the expression levels of proteases disintegrin and metalloproteinase 17 (ADAM17) and *TMPRSS2* were also significantly (*p* < 0.05) higher in obese patients (BMI > 30) relative to non-obese patients [83].

### 3.7. Gender

Sex-related severity and mortality appears to us as one of the most frequently reported data. According to an analysis of 239,709 patients in Italy, mortality was 17.7% in males and 10.8% in females, with 59% of the total deaths being males. However, the infection rate was lower in males than in females, with 45.8% and 54.2% of positive cases, respectively [108]. Devaux, et al. even stated that all clinical reports published up to that date indicated that men represented between 66% and 75% of the most severe cases of COVID-19 [109]. It should be noted that apart from severity and mortality, other studies have asserted that the incidence of COVID-19 is greater in males compared to females [90].

To understand how gender-related factors can be related to SARS-CoV-2 infection, it is important to keep in mind that multiple aspects may be at stake here simultaneously. Firstly, the conversion of Ang II to Ang (1–7) by ACE2 was higher in males than females, suggesting an overexpression of *ACE2* in men [89]. An analysis of disease outcomes in COVID-19 patients in two independent cohorts revealed a significant association between elevated free androgen and COVID-19 complications, pointing to a possible link between androgen-mediated *ACE2* regulation and disease severity [91]. Benign prostatic hypertrophy (BPH), a disease linked to elevated androgen levels, was independently associated with both COVID-19 susceptibility (OR 1.4; 95%CI, 1.2–1.8; *p* = 8.7 × 10^−4^) and COVID-19 hospitalization (OR 1.6; 95%CI 1.2–2.1; *p* = 2.2 × 10^−4^) in multivariate models adjusted for age, hypertension, type 2 diabetes, BMI, and the Townsend deprivation index. In particular, only 12.0% of the controls also had BPH while 17.9% of COVID-19 positive men and 21.2% of COVID-19 hospitalized men had BPH [91]. At the same time, treatment with antiandrogenic drugs modulated ACE2 and TMPRSS2 levels in both lung epithelial and cardiac cells and protected human embryonic stem cell (hESC)-derived lung organoids against SARS-CoV-2 infection [91].

Yee et al. also stated that *TMPRSS2* expression is highly regulated by androgens in the prostate gland, suggesting it may play a role in sex-dependent differences in the lung [92]. However it is important to keep in mind that, in other studies with mice or human models, no evidence was found for androgen regulation of TMPRSS2 in the male lung [88]. Still, even here, ACE2 and androgen receptor (AR) expression were higher in males than females [88]. Besides, awestern analysis of human airway smooth muscle cell lysates showed significantly higher *ACE2* expression in males compared to females at the baseline. Airway smooth muscle cells exposed to estrogen and testosterone for 24 h showed that testosterone significantly upregulates *ACE2* expression in both males and females, whereas estrogen (non-significantly) downregulates *ACE2* [90].

In conclusion, evidence from experimental studies clearly shows that higher levels of androgens in men directly upregulate the expression of the main receptors for SARS-CoV-2 and that would be the explanation for the greater severity of the disease observed in men. As a result, patients with prostate cancer that are treated with anti-AR therapy could be less susceptible to viral infection and the use of anti-AR compounds could be a therapeutic strategy and preventive option in these patients to avoid viral entry [110].

As a curiosity, in three different RNA expression databases, (Human Protein Atlas, FAMTOM5 and GETx), *ACE2* was found to be highly expressed in testicular cells at the protein levels, while little *ACE2* expression was seen in ovarian tissue [111]. The testes are a site of immune privilege, protected by the blood–testes barrier. As such, high *ACE2* expression combined with immune privilege may enable the testes to serve as viral reservoirs for COVID-19, leading to delayed viral clearance, potentially higher viral loads, and prolonged accumulative lung and systemic tissue damage [112]. However, throughout the evidence, this hypothesis seems unlikely to be the explanation for the effect.

### 3.8. Age

Advancing age is increasingly recognized as one of the strongest predictors for severe COVID-19 [113]. As with influenza and other respiratory viral infections, it is obvious that gradually decreasing innate and adaptive immune responses may be expected to play an important role in this age-related increased susceptibility [33]. However, *ACE2* expression in the lungs and SARS-CoV-2 viral load have been suggested to increase with age, which could also participate in the pathophysiology for the higher disease severity observed in older patients with COVID-19 [93]. In fact, hyperoxia-induced depletion of alveolar epithelial type 2 (AT2) cells (the major target of the virus in the lung) was expected to decrease the SARS-CoV-2 receptors *ACE2* and *TMPRSS2* expression in the lung. Instead, the expression of *ACE2* and *TMPRSS2* mRNA was found to increase with the age of mice and this was accelerated by exposing mice to neonatal hyperoxia. This serves as a protective mechanism as the lung ages to preserve AT2 cells and thus reduce or prevent the development of idiopathic pulmonary fibrosis [92]. These findings are in agreement with a review that discussed two unpublished studies showing how the expression of *ACE2* and *TMPRSS2* mRNA increases with age in the human respiratory epithelium [94].

Thus, an age-dependent increase in SARS2-CoV-2 receptors in the respiratory epithelium may be also responsible for the increased severity of COVID-19 lung disease in elderly people. Regarding children, research reports showed the *ACE2* gene expression in nasal epithelium was significantly higher in older children (10 to 17 years old), young adults (18 to 25), and adults (more than 25 years old) when compared with younger children (less than 10 years old) [114]. Moreover, preterm and term newborns have a lower expression of *ACE2* in the nasal epithelium than adults [115]. This may explain why COVID-19 is less prevalent and severe in children [116].

Regarding SARS-CoV-2 variants, a cohort study that enrolled 839,278 patients with COVID-19 showed that 27,720 were hospitalized with the B.1.1.7. variant and these patients were associated with a more severe disease, especially in adults older than 30 years old [117]. 

### 3.9. Hypertension

As discussed earlier, hypertension is one of the main comorbidities associated with worse outcomes in COVID-19. A pooled analysis of the current scientific literature suggested that hypertension may be associated with an up to 2.5-fold higher risk of severe and fatal COVID-19 [118]. Thus, it would be relevant to explore the expression of *ACE2* in hypertensive patients relative to non-hypertensive patients, but, in our literature review, scientific evidence concerning hypertension and COVID-19 focused almost exclusively on the possible effects of RAAS inhibitors (commonly prescribed drugs for various indications, such as hypertension, myocardial infarction, cardiac failure, kidney diseases, and complications of diabetes all over the globe) and not on the purpose of our review.

### 3.10. Renin-Angiontensin-Aldosterone System Inhibitors

The use of RAAS inhibitors was under the controversial discussion of possible deleterious versus possible protective effects. In the “deleterious” hypothesis, RAAS inhibition would lead to the upregulation of *ACE2* expression at the cell surface, which would promote SARS-CoV-2 entry. In the “protective” hypothesis, RAAS inhibition would decrease the formation of angiotensin II, offering protection from inflammation and fibrosis in the lung [44]. ARBs would even lead to competition with Ang II for AT1R, resulting in increased Ang II to be processed by ACE2 and increased Ang (1-7) levels, with vasodilation and anti-fibrotic effects. Furthermore, the binding of ACE2 to circulating Ang II could induce a conformational change resulting in a less favorable binding of SARS-CoV-2 to its receptor and the decreased internalization of the virus [33].

Currently, it has been proved that there is no association between the exposure to RAAS inhibitors and the risk and severity of COVID-19 infection, supporting the current medical guidelines and recommendations that patients should not discontinue RAAS inhibitors [95,96,97,98,119,120].

## 4. Conclusions

The COVID-19 pandemic has caused tremendous health burdens worldwide and has had a tremendous economic impact. In this article, we reviewed some significant aspects of the SARS-CoV-2 invasion that are proving to be complex and difficult to tackle and dwelt on how several factors may interfere with the virus main host cell receptor, ACE2, which is intrinsically related to the damage that arises upon infection.

Higher levels of ACE2 expression in patients with comorbidities such as cardiovascular disease, COPD, and in diabetic pancreatic islets increase the susceptibility of contracting SARS-CoV-2 infection and subsequent COVID-19 severity. *ACE2*, which is also highly expressed in adipose tissue, and higher levels of androgens in men were shown to directly upregulate the expression of *ACE2*. Age-dependent increases in SARS2-CoV-2 receptors in the respiratory epithelium may be also responsible for the increased severity of COVID-19 lung disease in elderly people. Finally, IL-13 cytokine decreases the expression of *ACE2* in the nasal and airway epithelial cells of asthmatic type 2 patients, which can explain the absence of increased risk in this group of patients, typically associated with respiratory viral infections exacerbations. Further studies are needed to show a direct correlation between ACE2 and TMPRSS2 expressions in cancer patients and the incidence and severity of COVID-19.

We hope this review has shed light on this subject and will help encourage scientific efforts with further experimental studies to analyze aspects of ACE2 in relation to COVID-19 pathophysiology. This is paramount to fuel the development of future therapeutic strategies, such as the manipulation of *ACE2* expression in target tissues of SARS-CoV-2.

## Figures and Tables

**Table 1:** Correlation between *ACE2* expression and comorbidities/risk factors associated with COVID-19 severity.

Comorbidities/Risk Factors	Conclusion	References
Cardiovascular Disease	Increased myocardial *ACE2* expression (pericytes).	[16,47,69,70,71]
Respiratory Conditions: COPD and Lung Cancer	Increased airway/lung tissue *ACE2* expression.	[59,72,73,74,75,76,77,78,79]
Obesity	Expression levels of *ACE2* in adipose tissue higher than those in the lung.	[7,33,80,81,82]
Diabetes	Increased pancreatic islets *ACE2* expression.	[83,84]
Asthma (type 2) ^#^	IL-13 down-regulates *ACE2* expression in the nasal and airway epithelial cells.	[85,86,87]
Male gender	Androgens regulate the transcription of the *ACE2* gene, up-regulating its expression at the cell surface.	[88,89,90,91,92]
Age	*ACE2* expression seems to increase with age in the respiratory epithelium.	[92,93,94]
RAAS Inhibitors	Not associated with increased risk in COVID-19 patients.	[95,96,97,98]

**^#^** not a comorbidity with higher risk in COVID-19, as it could be expected.

## Data Availability

Not applicable.
